# Mycobacteria infect different cell types in the human lung and cause species dependent cellular changes in infected cells

**DOI:** 10.1186/s12890-016-0185-5

**Published:** 2016-01-23

**Authors:** Dariimaa Ganbat, Sophie Seehase, Elvira Richter, Ekkehard Vollmer, Norbert Reiling, Kurt Fellenberg, Karoline I. Gaede, Christian Kugler, Torsten Goldmann

**Affiliations:** Clinical and Experimental Pathology, Research Center Borstel, Borstel, Germany; Mongolian National University of Medical Sciences, Ulaanbaatar, Mongolia; Airway Research Center North (ARCN), Member of the German Center for Lung Research, Gießen, Germany; National Reference Center for Mycobacteria, Research Center Borstel, Borstel, Germany; Microbial Interface Biology, Research Center Borstel, Borstel, Germany; Bioinformatics, Research Center Borstel, Borstel, Germany; Thoracic Surgery, Lungen Clinic Grosshansdorf, Grosshansdorf, Germany; Present address: Labor Limbach, Heidelberg, Germany

**Keywords:** Tuberculosis, Ex vivo, STST model, Mycobacteria, *M. tuberculosis*, *M. avium*, *M. abscessus*, Cellular change, HOPE-fixation, Innate immunity, Intracellular infection, Tissue viability

## Abstract

**Background:**

Mycobacterial infections remain a significant cause of morbidity and mortality worldwide. Due to limitations of the currently available model systems, there are still comparably large gaps in the knowledge about the pathogenesis of these chronic inflammatory diseases in particular with regard to the human host. Therefore, we aimed to characterize the initial phase of mycobacterial infections utilizing a human ex vivo lung tissue culture model designated STST (Short-Term Stimulation of Tissues).

**Methods:**

Human lung tissues from 65 donors with a size of 0.5–1 cm^3^ were infected each with two strains of three different mycobacterial species (*M. tuberculosis, M. avium,* and *M. abscessus*), respectively. In order to preserve both morphology and nucleic acids, the HOPE® fixation technique was used. The infected tissues were analyzed using histo- and molecular-pathological methods. Immunohistochemistry was applied to identify the infected cell types.

**Results:**

Morphologic comparisons between ex vivo incubated and non-incubated lung specimens revealed no noticeable differences. Viability of ex vivo stimulated tissues demonstrated by TUNEL-assay was acceptable. Serial sections verified sufficient diffusion of the infectious agents deep into the tissues. Infection was confirmed by Ziel Neelsen-staining and PCR to detect mycobacterial DNA. We observed the infection of different cell types, including macrophages, neutrophils, monocytes, and pneumocytes-II, which were critically dependent on the mycobacterial species used. Furthermore, different forms of nuclear alterations (karyopyknosis, karyorrhexis, karyolysis) resulting in cell death were detected in the infected cells, again with characteristic species-dependent differences.

**Conclusion:**

We show the application of a human ex vivo tissue culture model for mycobacterial infections. The immediate primary infection of a set of different cell types and the characteristic morphologic changes observed in these infected human tissues significantly adds to the current understanding of the initial phase of human pulmonary tuberculosis. Further studies are ongoing to elucidate the molecular mechanisms involved in the early onset of mycobacterial infections in the human lung.

## Background

Mycobacterial infections are still a major cause of morbidity and mortality worldwide. In 2013 (WHO) an estimated number of 9.0 million people developed Tuberculosis (TB) and 1.5 million people died of this chronic inflammatory disease [1]. It is currently not clear whether the comprehensive strategies developed by the WHO will lead to the elimination of this infectious disease by 2050. Independently, nontuberculous mycobacterial (NTM) infections mainly caused by *M. avium, M. intracellulare, M. abscessus*, and *M. chelonae* are increasing [2]. NTM are ubiquitous in the environment and can cause a wide range of infections in humans, as well as in animals [3]. Many different animal models have been developed in mycobacterial research since Robert Koch’s period. However, existing models have so far failed to mimic human disease. Major disadvantages include significant differences in mycobacteria-induced pathology and relative resistance (mice and rats), high costs (non-human primates), or different immunological capacities compared to humans (guinea pigs and rabbits) [4–8]. Thus the lack of appropriate models for basic research in mycobacterial infection of the human host hampers new insights into disease mechanisms and scientific progress with regard to successful measures to accomplish that goal [9].

Various in vitro models with human cells have been established. However the results obtained by different research groups are often hard to compare [10] due to the use of different bacterial strains and infection doses [11, 12] and the large differences in study design including different (i) cell types (monocytes, macrophages, neutrophils, and microglia [13–16]), (ii) cell lines (macrophage-like cells and non-phagocytic cell lines [17–21]), (iii) host cell sources (human, healthy or patients, and animals [22–24]), and (iv) last but not least incubation media (containing supplements or not [25, 26]). In addition, most of the gathered information indicates that it is extremely difficult to induce mycobactericidal activity in purified populations of phagocytes. Therefore, some more complex models have been developed, e. g. co-culture of immune cells [13], whole blood assays [27], or microenvironments comprising epithelial and endothelial cells [28], as well as the use of distinct stimuli (e.g. cytokines, vitamins, lipids, and nucleotides [29–32]). Nonetheless, these large variations of results do not allow definite conclusions.

From a host perspective it needs to be mentioned that 50 % of individuals exposed to *M. tuberculosis* (Mtb) never become tuberculin skin test positive, which may indicate that the mycobacterium is removed by the innate immunity [33]. Likewise, there are several lines of epidemiological evidence supporting a protective role for innate immunity in tuberculosis. The successful elimination of pathogenic mycobacteria early on during infection by the innate immune-system is still controversially discussed and very likely underestimated due to the lack of human studies.

In order to study early innate effector mechanisms upon mycobacterial infection and on the current lack of complex human-relevant models, an ex vivo tissue culture model, referred as STST (Short-Term Stimulation of Tissues), was developed. The main advantage of this lung tissue model is the maintenance of the intact lung microenvironment with its native cell population, orientation, and structural integrity. The STST model of human lung tissue has been successfully used to obtain valuable information about early steps in the pathogenesis of several infectious lung diseases, including infections with *Legionella pneumophila, Pseudomonas aeruginosa, Streptococcus pneumonia, Chlamydia pneumoniae*, and *Haemophilus influenzae* [34–38].

## Methods

### Ethical statement and collection of samples

Human lung tissue specimens were acquired from surgical material of 65 patients, who underwent pneumonectomy or lobectomy due to cancer at the LungenClinic Grosshansdorf, Germany. The study was performed with permission of the local ethical committee at the University of Lübeck, written informed consent was obtained (Approval number: 07–157).

### Bacterial strains and culture

Following strains were used for the study at different colony forming units (CFU)/ml (10^4^–10^7^), which were cultivated in Löwenstein–Jensen medium (LJ): *M. abscessus* 9547/00 (type strain, =AB1), *M. abscessus* 8562/11 (clinical isolate, =AB2), *M. avium* 3725/07 (strain 104, =AV2), *M. avium* 3439/10 (clinical isolate, =AV1), *M. tuberculosis* 9679/00 (type strain H37Rv, =TB2), and *M. tuberculosis* 1616/12 (clinical isolate from a German patient, =TB1). In order to precipitate mycobacterial clumps, suspensions were centrifuged at low speed (100 × g) for 5 min. BBL™ MGIT™ PANTA™ antibiotic mixture (BD diagnostics, USA) was added to the suspensions to prevent other bacterial growths. The concentrations of viable mycobacteria (CFU/ml) in the stock suspensions were controlled three times during the study. Basically, the stock solutions were serially diluted (1:10 each) until 10^0^ CFU/ml. From 10^0^ to 10^3^ CFU/ml 0.3 ml were cultured in petri dishes containing LJ medium. Cultivation time for *M. abscessus* was 1–2 weeks and for *M. avium,* as well as *M. tuberculosis* 4–6 weeks, respectively. The mycobacterial colonies were counted visually and the CFU/ml were determined. For infection of the lung tissue specimens, 2 ml of suspension from each strain were used.

### Infection of the lung tissue specimens

Under gross morphologic examination, only parts of the surgical materials without inflammatory consolidations, pleura, neoplasia, or anthracosis, were selected and dissected with a size of 0.5–1 cm^3^ (~30 mg) for the investigation. In order to optimize the viability of the tissues in the ex vivo system, as well as to determine the best suitable amount of tissues and volume of culture medium, MTT assays [(4,5-dimethylthiazol-2-yl)-2,5-diphenyltetrazolium bromide] have initially been performed (data not shown). According to these, each piece of tissue was infected with 2 ml of the suspensions. To distribute the mycobacteria, medium was gently re-suspended by pipetting. Infection and cultivation of tissues were performed for 16 h in RPMI1640 (1×) GlutaMAX™-I (Invitrogen, Darmstadt, Germany) supplemented with 10 % FCS (PPA, Pasching, Austria), 0.02 M HEPES buffer solution (Life technologies, Invitrogen, Carlsbad, California, USA), and 0.01 mM sodium pyruvate (Biochrom, Berlin, Germany) at 37 °C overnight in 24-well, flat bottom Corning® Costar ® tissue culture plates (Sigma-Aldrich Co., St. Louis, USA). Titration experiments were initially carried out to select optimal infection dose of mycobacteria, where the highest number of infected cells was obtained. Thus a CFU/ml of 10^7^ was used for all experiments. Additionally, two kinds of control tissues were included, one was freshly fixed without cultivation, and another was incubated under the same culture conditions without mycobacterial infection (medium control).

### Tissue processing and histopathological analysis

HOPE® fixation technique (HEPES-glutamic acid buffer mediated Organic solvent Protection Effect) (DCS Diagnostics, Hamburg, Germany) was used as previously published [39, 40]. There were no washing steps for the ex vivo infected tissues before fixation. Briefly, specimens were fixed in HOPE® solution at 4 °C overnight. Dehydration procedure was performed with 100 % acetone at 4 °C and repeated 4 times. Tissue samples were directly embedded in low-melting paraffin (DCS *lab*Line, Germany). Tissue blocks were cut on a microtome, sections were deparaffinized with isopropanol (2×10 min at 60 °C) and subsequently visualized by histological stainings depending on purposes: 1) Hematoxylin and Eosin (H&E) staining, allowing a general morphological inspection after ex vivo incubation (Merck, Darmstadt, Germany), 2) Gram staining for the detection of gram-positive bacterial contamination (Merck, Darmstadt, Germany), 3) cold Ziel Neelsen (ZN) technique/Kinyoun staining for the detection of mycobacteria (bioMérieux SA, Craponne, France).

### IHC and fluorescence double staining

Double-staining was performed comprising IHC (to identify cell types) with Auramine-Rhodamine fluorescence staining (to detect mycobacteria) (Waldeck GmbH & Co. KG, Münster, Germany). For immunohistochemical staining, following antibodies were used depending on the purpose. Herein: Anti-Human CD 68 for macrophages (diluted 1:200, clone: PG-M1, monoclonal, mouse, Dako Cytomation, Glostrup, Denmark), Anti-Human Neutrophil Elastase (NE) for neutrophils (diluted 1:200, clone: NP57, monoclonal, mouse, Dako Cytomation, Glostrup, Denmark), Anti-Surfactant Protein-C (SP-C) for pneumocytes-II (diluted 1:300, clone: FL-197, polyclonal, rabbit, Santa Cruz Biotechnology Inc., Dallas, Texas, U.S.A.), for lymphoid cells Anti-Human T cell, CD8 (diluted 1:50, clone: C8/144B, monoclonal, mouse, Dako Cytomation, Glostrup, Denmark), Anti-Human CD20cy, B cell (diluted 1:100, clone: L26, monoclonal, mouse, Dako Cytomation, Glostrup, Denmark), Anti-human CD30, (diluted 1:50, clone: Ber-H2, monoclonal, mouse, Dako Cytomation, Glostrup, Denmark), Anti-Human T cell, CD4 (diluted 1:100, clone: MT310, monoclonal, mouse, Dako Cytomation, Glostrup, Denmark), and Anti-Human CD79a (diluted 1:200, clone: SP18, monoclonal, rabbit, DCS Innovative Diagnostik-System GmbH & Co, Hamburg, Germany). For detection, the ZytoChemPlus (HRP) Polymer kit (Zytomed Systems, Berlin, Germany) was applied with above mentioned antibodies and Diaminobenzidine (DAB) as substrate. After IHC staining, the slides were subsequently transferred to Auramine-Rhodamine dye (15 min), HCl- Alcohol 70 % (2 min) and potassium permanganate (KMnO_4_) 0.5 % (2 min 30 s) in the dark. Examination and capture of images were carried out by a fluorescence microscope (Eclipse 80i, Nikon). In order to provide simultaneous detection of double staining, the same area of fluorescent photomicrographs of the fluorochrome (Rhodamine/TRITC, spectral characteristics: 550–580) and color inverted brightfield-images of the IHC detection were overlaid (FixFoto).

### Measurements of cellular components

The following parameters were measured: 1) Cytoplasmic and nuclear diameters, 2) Light densities (LDs) of cytoplasm and nucleus. Then, N/C ratio (a ratio of the nuclear size to the cytoplasmic size) and arithmetic mean of nuclear and cytoplasmic LDs were estimated. All measurements were performed with the Infinity analyze software after background correction. To keep measuring light densities of cells constant camera control options were kept at a magnification of 400×, exposure time = 200 ms, gain = 2, gamma = 1, color correction matrix/light source = daylight.

### Viability test

TUNEL (Terminal deoxynucleotidyl transferase dUTP nick end labeling) assay was performed in HOPE-preserved tissue sections. The slides were initially incubated for 15–30 min at 37 °C with proteinase K and rinsed twice with PBS buffer. After adding TUNEL reaction mixtures, the slides were incubated at 37 °C in humidified atmosphere for 1 h and again rinsed 3 times with PBS buffer. All procedures were carried out in a dark environment. Freshly HOPE-fixed lung tissues served as positive control (alive), whereas tissues treated by DNase I served as negative control. For counter staining Vectashield mounting medium with 4′–6-diamidino-2-phenylindole (DAPI) was used (Vector Laboratories, Burlingame, USA). Examination and capture of images were performed by a fluorescence microscope (Eclipse 80*i,* Nikon). Cell counts were acquired using Infinity Analyze software after adjustment of linear contrast. With a purpose of analyzing viability, integrated measurements of all ex vivo incubated samples were compared with positive (untreated lung specimens) and negative controls (DNase I treated lung specimen).

### Software and statistics

FixFoto (version 3.20, Joachim Koopmann Software, Wrestedt/Stederdorf, Germany), Image J (version 1.48b, Wayne Rasband, Maryland, USA), Infinity analyze (version 6.0.02, Lumen*era* corporation, Ontario, Canada) were used for optimizing micrographs. IBM SPSS (version 20.0.0, IBM Corporation, New York, United States) and Graph Pad Prism 6 (version 6.04, GraphPad Software, Inc. California, USA) were used for statistical analysis. Statistical tests were chosen as indicated in the figure legends. A p-value <0.05 was considered statistically significant. Correspondence analysis was performed using MATLAB (MathWorks, Inc., Natick, MA, USA) as described [41].

## Results

### The STST model in the analysis of mycobacterial infections

**Fig. 1:**
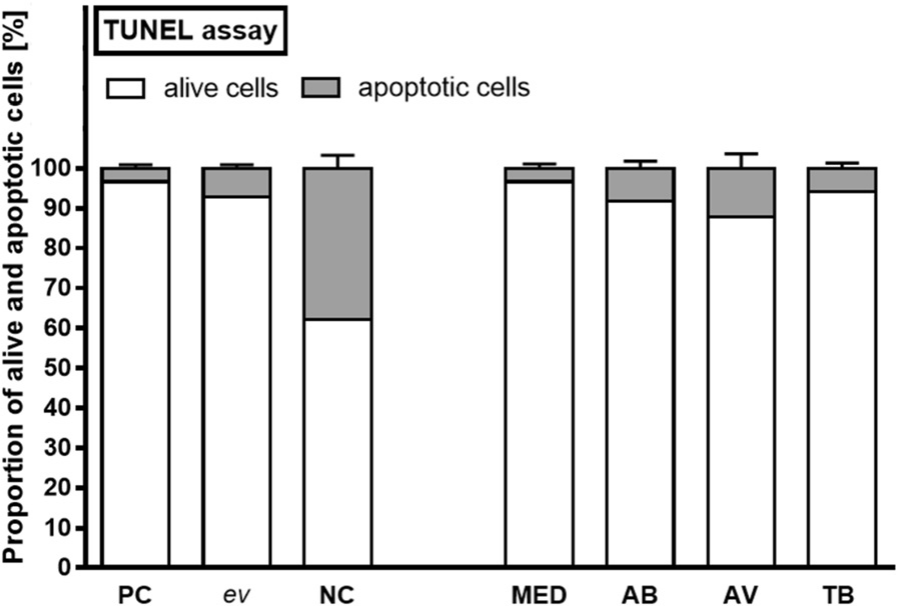
Results of the TUNEL assays. PC = Positive Control (fresh lung preparations without ex vivo cultivation, *N* = 17), *ev*: Mean of all different *ex vivo* cultivated lung specimens, including Medium control (MED, *N* = 12), lungs infected with *M. abscessus* (AB, *N* = 17), *M. avium* (AV, *N* = 7) and *M. tuberculosis* (TB, *N* = 18). NC = Negative Controls (treated by DNase I, *N* = 8). Left panel: No significant increase of DNA-double strand breaks upon tissue culture. Right panel: Differences between the respective mycobacterial species. These results related with multiple factors analyzed by a factorial ANOVA [2×6 factorial design: subject factors were cell type (1-alive and 2-apoptotic cells) and groups (1-PC, 2-MED, 3-AB, 4-AV, 5-TB, 6-NC)]

The morphology of all incubated tissues (in the absence or presence of mycobacteria) was explored and compared to non-incubated, freshly fixed lung tissues by H&E staining. No noticeable differences were detected among the collected specimens at tissue level. The viability of tissues after incubation was evaluated by TUNEL assay, which detects DNA double-strand breaks during apoptosis, allowing the quantification of mycobacteria dependent cell death (Fig. 1 and Table 1). Altogether, 96.64 ± 3.65 % of cells in the positive controls (fresh lung preparations without ex vivo cultivation) were viable compared to 62.09 ± 11.95 % in the negative controls (treated by DNase I). The ex vivo STST samples revealed 92.85 ± 6.99 % of living cells in total, while 7.15 ± 6.99 % were apoptotic as demonstrated by positive staining for TRITC.

**Table 1:** Influence of the ex vivo culture on tissue viability (Counting analysis of TUNEL assay)

Groups	Number of cells (M ± SD)		Area of cells (M ± SD) [mm^2^]	
	Alive	Dead	Alive	Dead
Positive control	383.75 ± 106.67	11.88 ± 10.87	64.76 ± 32.54	8.30 ± 1.05
Negative control	284.38 ± 150.75	167 ± 84.03	49.13 ± 32.06	14.62 ± 8.664
ex vivo sample	366.04 ± 127.50	27.43 ± 27.46	52.94 ± 31.07	1.73 ± 1.90

**Fig. 2:**
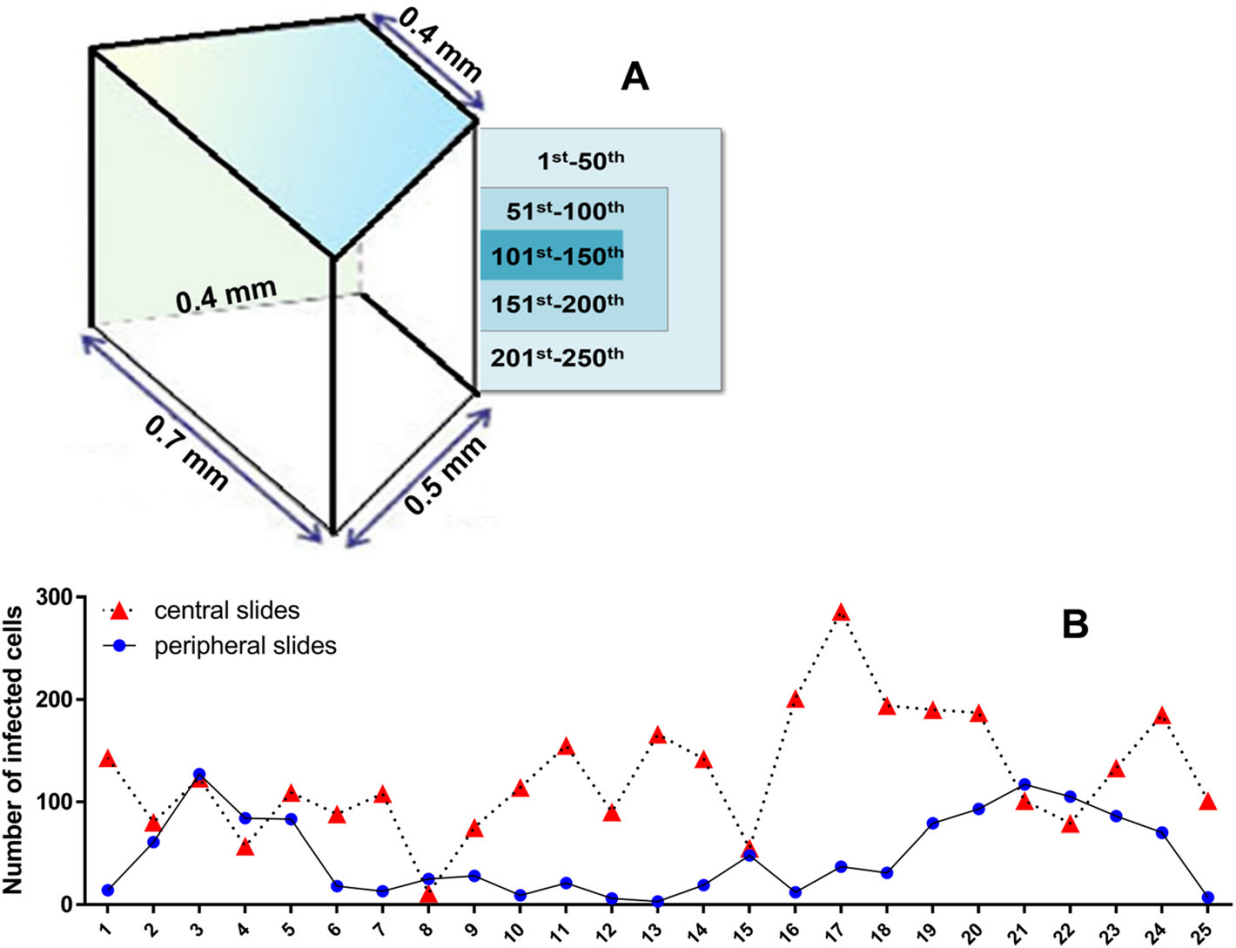
**a** Observation of serial sections of a whole paraffin block. Its 3D trapezoid size was approximately 0.7×0.5×0.4×0.4 mm. Serial cuts resulted in 250 sections with a thickness of 2 μm. The 1st -50th and 201st -250th sections were considered to be identical (same marginal condition), as well as the 51st -100th and 151st -200th sections (same para-peripheral condition). To compare incidents of infected cells among tissue, every odd numbered slides from 1st-50th (peripheral region, N = 25) and 101st-150 (central region, N = 25) were utilized. **b** Distribution of infected cells in different layers of the tissue. The x-axis shows the number of sections, the y-axis the numbers of infected cells. Descriptive statistics showed that the peripheral slides were associated with a number of infected cells 47.87 ± 38.9, whereas, central slides had 126.92 ± 59.47 of infected cells with high variances, followed by inferential statistics (non-parametric Mann–Whitney test)

In order to detect mycobacterial infections and also other possible bacterial contaminations, all samples were stained by Ziehl Neelsen (ZN) and Gram-staining in parallel. Consequently, all cases of ex vivo infected STST-samples were ZN-positive while Gram-stainings remained negative. For verification of mycobacterial infection in the human ex vivo tissues, specimens were screened for the detection of mycobacterial DNA by PCR (Polymerase chain reaction). Mycobacterial DNA was not detected in medium controls (negative), whereas samples from the infected tissues were positive with regards to the mycobacterial species used for infection (data not shown). Confirmation of infecting strains was performed by DNA-sequencing of the PCR-products. Since mycobacteria are non-motile, we measured how deep the bacilli infiltrate in a given tissue. To this end, serial sections of one whole tissue block were prepared and analyzed by ZN-staining (Fig. 2a). Statistical analysis by Mann–Whitney *U*-test revealed the mean rank of infected cells on the peripheral (*Mrank = 16.38*) to be even significantly lower than the mean rank of infected cells on the central slides (*Mrank = 34.62*), U = 84.5, Z value = −4.424, *p* < 0.0001 (Fig. 2b), demonstrating a sufficient infiltration of the bacteria into the tissues.

### Mycobacteria infect different cell types in the human lung

**Fig. 3:**
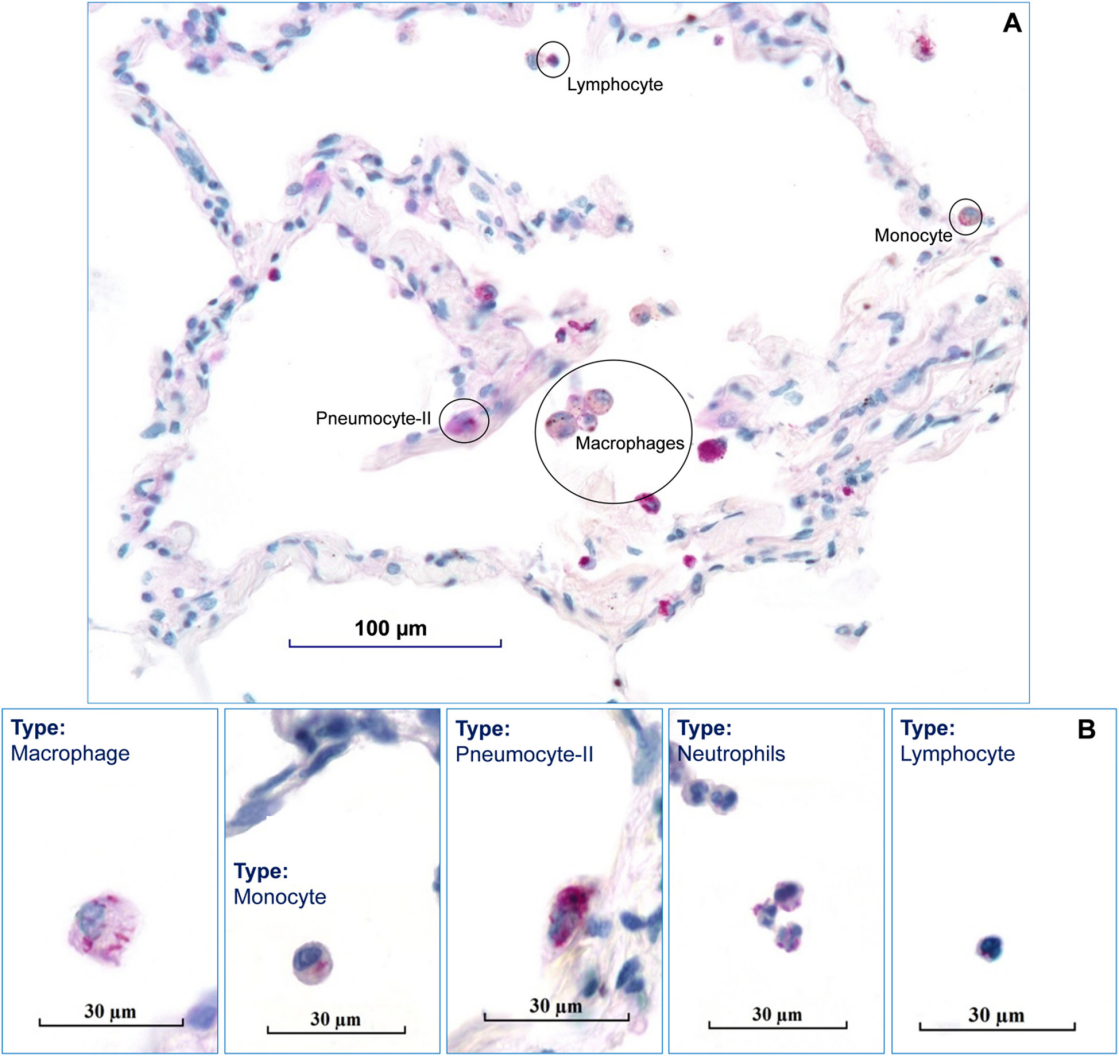
Mycobacteria are taken up by different cell types in the *ex vivo* infected human lung tissues **a**. Infected cell with labels, at × 640 magnification, intracellular bacilli were visualized by Ziel Neelsen staining in pink color, scale bar =100 μm. **b**. Infected cell types were shown separetely with labels, the images were acquired at × 1000 magnification, scale bars = 30 μm

**Fig. 4:**
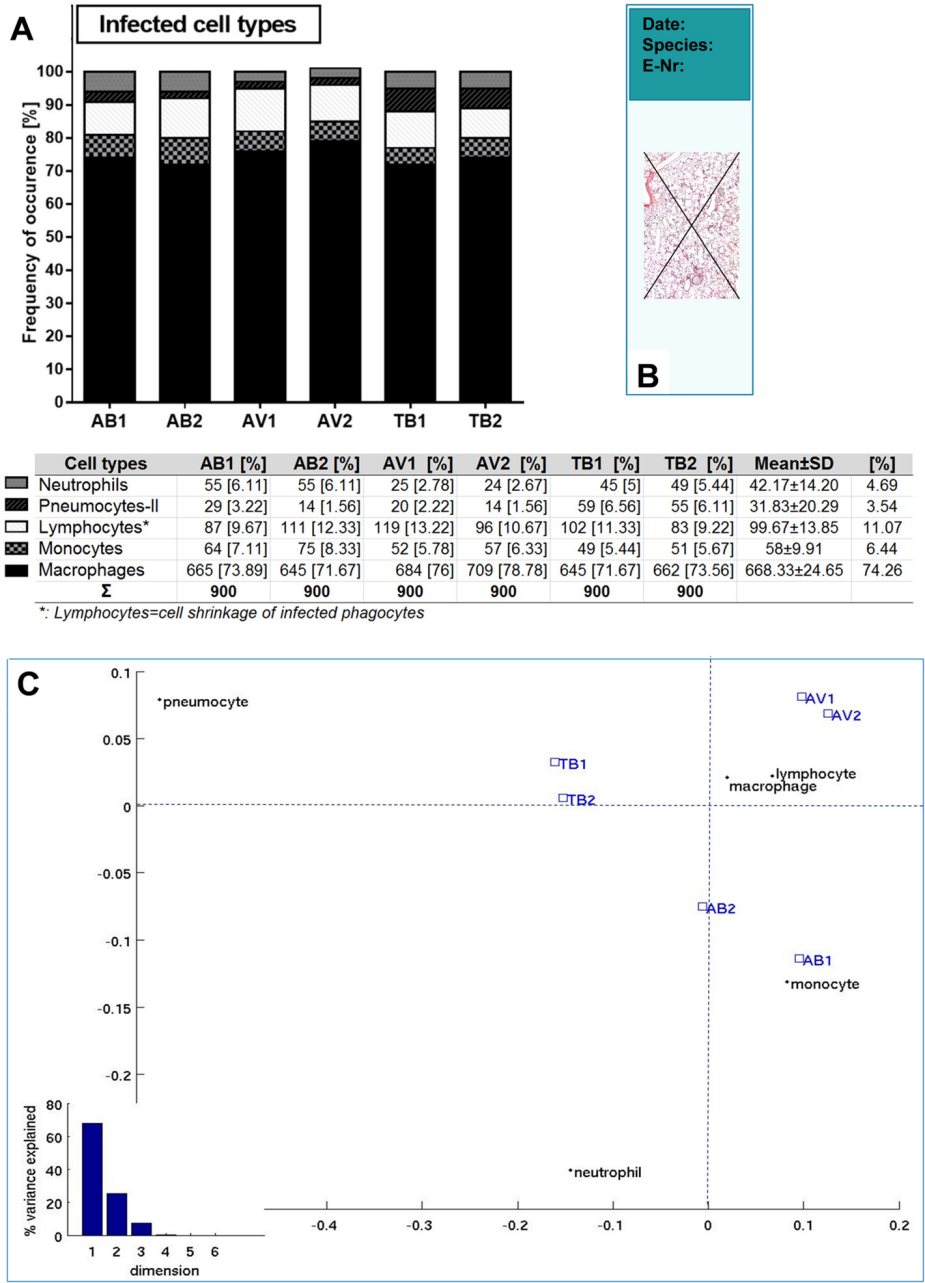
Cellular populations infected by 6 mycobacterial strains. **a** The frequencies of the infected cell types shown in the graph and occurrences of absolute numbers given in the table. **b** 30 infected cells were counted among 2 diagonal lines in each slide and in order to involve whole tissue equivalently as illustrated. **c** Correspondence analysis map for mycobacterial strains and cell types. Dispersions of all profile points produced an asymmetric map. The centroid was labeled by a star. Around the centroid, the profile points of the various mycobacterial species (blue square) and of the different cell types (black dot) were plotted. A bar plot of variances is shown at the left bottom part of the map

Microscopical analysis of the prepared sections with high magnification (400×) identified different cell types to be infected with mycobacteria (Fig. 3a and b) including macrophages, neutrophils, monocytes, cells morphologically looking like lymphocytes, and pneumocytes-II. To characterize the frequency of the infected cell types, 30 infected cells were counted from randomly chosen 30 donors’ sets of specimens (Fig. 4a and b), containing 6 mycobacterial strains, which means that all in all 30×30×6 = 5400 infected cells were counted. Experiments using independent strains of the same species lead to similar results. A total of 4.69 % [42.17 ± 14.20] of the infected cells were identified neutrophils, 3.54 % [31.83 ± 20.29] were pneumocytes-II, whereas cells with a small cytoplasm account for 11.07 % [99.67 ± 13.85]. Monocytes were 6.44 % [58 ± 9.91], while macrophages were the most frequently infected cells with 74.26 % [668.33 ± 24.65].

The percentage of infected pneumocytes-II and neutrophils showed significant differences depending on the mycobacterial species used (*p* < 0.05, Kruskal-Wallis test), with pneumocytes-II being more infected with the TB-strains compared to AV and AB, and the AB-strains being more present in neutrophils respectively. Other cell types showed comparable infection rates, such as cells morphologically appearing like lymphocytes (*p* = 0.683), monocytes (*p* = 0.761), and macrophages (*p* = 0.368). A correspondence analysis visualizing the susceptibility of the cell types to certain mycobacterial strains is shown in Fig. 4c. Strains belonging to the same species are plotted adjacent to each other due to commonalities of the strains. Macrophages and lymphocyte-looking cells are located near the plot center, showing no special association with any strain. In other words, infections of these two cell types were similar among the 6 strains. By contrast, infected pneumocytes-II were significantly more abundant after infection with TB strains. Neutrophils and monocytes were more abundant after AB infections.

### Morphometric considerations reveal an even higher proportion of primarily infected cells that are not macrophages/monocytes

**Fig. 5:**
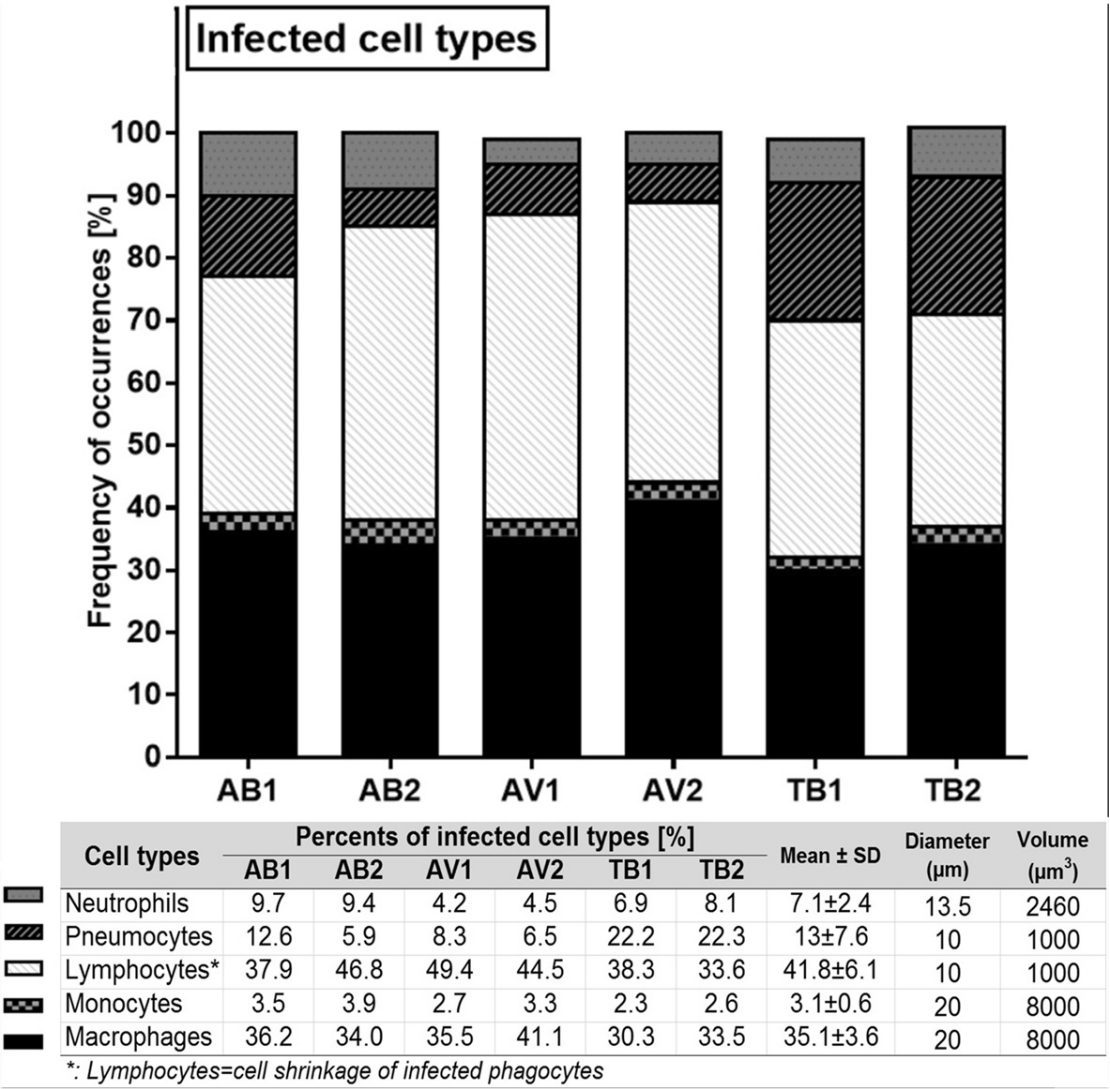
Factual proportions of infected population of cells among the 6 mycobacterial strains. The used cellular parameters for adjustments are included

The detection of a cell in these sections linearly depends on the volume of the cell under the assumption of an approximately round cell shape. The described cell types have different sizes and cellular volumes (Fig. 5), which affect the detection in the system used here (cutting, ZN-staining). Since the volume is the third dimension of the size (cell length), comparably small differences in the size largely affect the detection. In order to at least speculate about these relations in vivo, we adjusted the detection rates according to the different cellular volumes (Fig. 5, lower table).

After this correction, about 1/3 of cells infected by all mycobacterial species were macrophages, (35.10 ± 3.59 %), whereas neutrophils and monocytes account for 7.14 ± 2.38 and 3.05 ± 0.63 %, respectively. The percentage of infected pneumocytes-II was 12.96 ± 7.55 % with high variance. Interestingly, this cell type was more frequently infected by TB than by any other mycobacterial species analyzed (22.22 % versus 7.40 % [AV] and 9.27 % [AB]). The major group of infected cells showed a small cytoplasm, morphologically appearing like lymphocytes (41.75 ± 6.06 %). This was observed regardless of the mycobacterial species used.

**Fig. 6:**
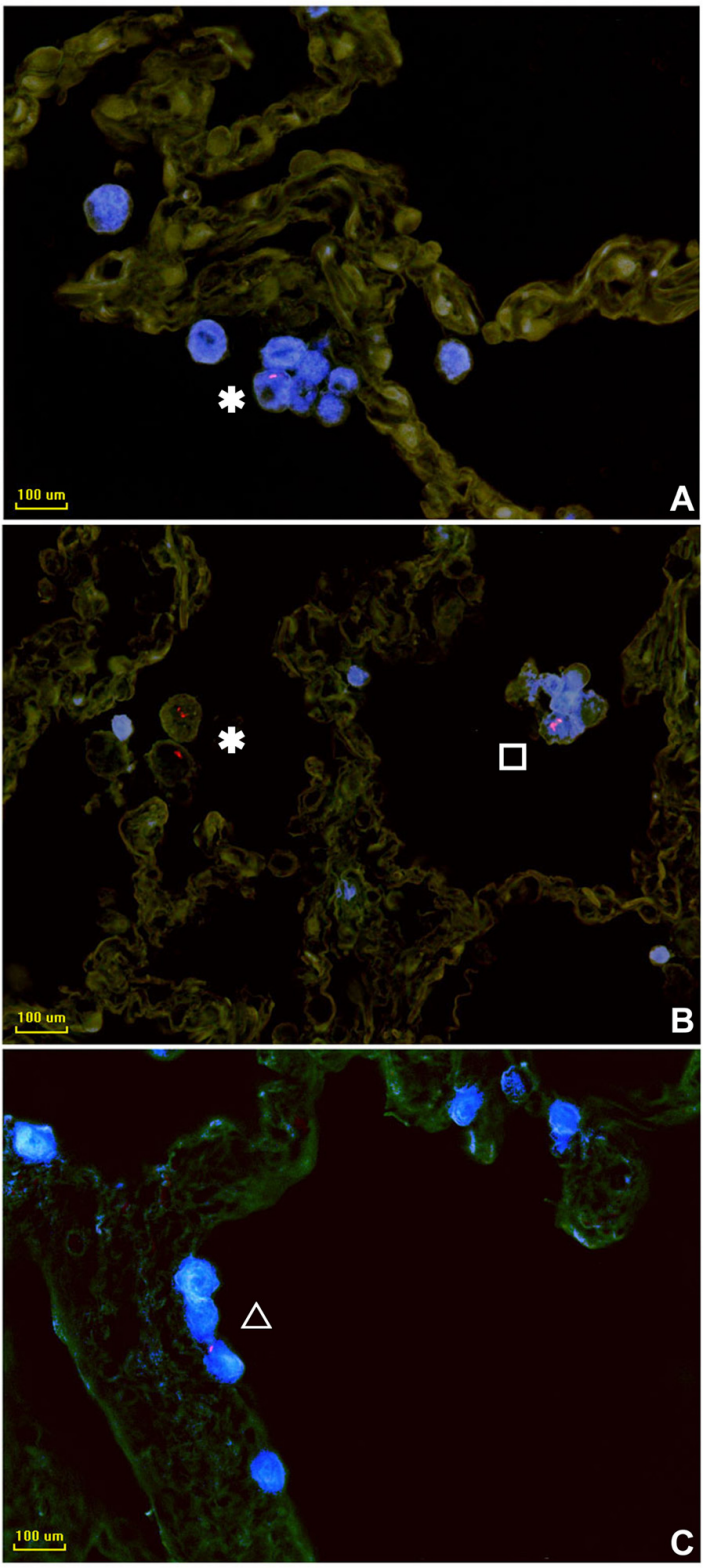
Photomicrographs of intracellular mycobacteria detected by double staining. **a** Macrophages (*), **b** Neutrophils (□), **c** Pneumocytes-II (∆).Slides stained by combination of auramine-rhodamine (for mycobacteria) and IHC of CD 68 (macrophages), NE (neutrophils) and SP-C (pneumocytes-II), respectively. The fluorescence signals of mycobacteria are red. Brightfield was used to capture immunohistochemical detections. Images were color-inversed to negative form which is why brown color of the color-substrate diaminobenzidine was shifted to blue. Overlays of the fluorescent and negative images were performed by using FixFoto. All images were acquired at × 400 magnification, scale bar =100 μm

In order to prove the identity of infected cell types, double-stainings of immunofluorescence and IHC were carried out (Fig. 6). Consequently, double-stainings allowed molecular identification of infected cell types, including neutrophils (NE), macrophages (CD68), and pneumocytes-II (SP-C). Due to the lack of suitable antibodies, the discrimination of macrophages and monocytes has not been performed. Interestingly, the morphologically detected cells with a small cytoplasm, which show a lymphocyte-like appearance (s. above) remained negative with all applied antibodies (including CD8, CD20, CD30, CD4, CD79), suggesting that these cells are not of lymphoid origin but could have undergone a progressive cellular shrinkage upon infection.

### Cell injuries induced by mycobacteria

**Fig. 7:**
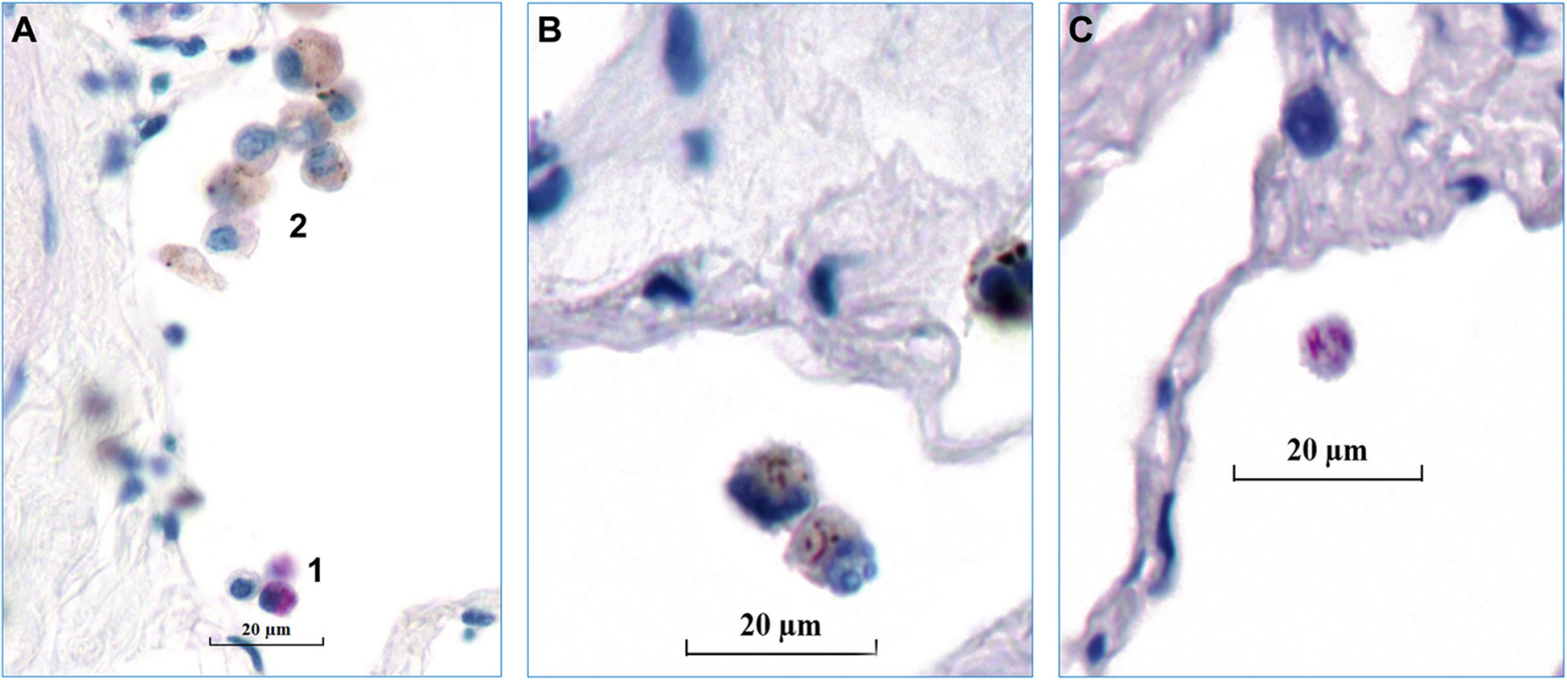
Cellular changes in infected cells. Intracellular bacilli were visualized by Ziel Neelsen staining and appear in pink color, scale-bars = 20 μm. **a** Cell schrinkage of infected macrophages (1) and normal macrophages (2), ×680 magnification. **b** Karyorrhexis of an infected macrophage (×1000). **c** Karyolysis of an infected macrophage (×1000)

**Fig. 8:**
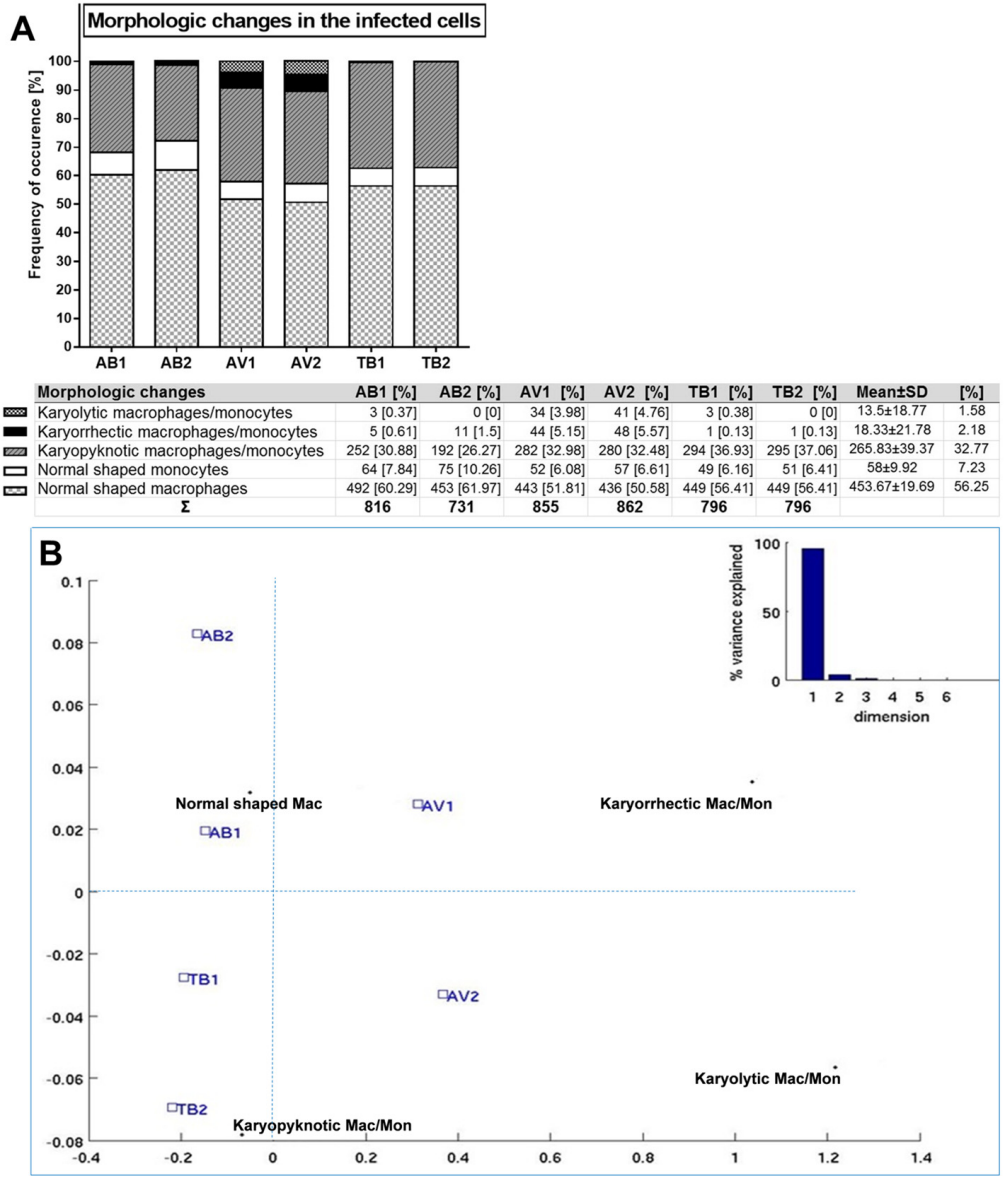
Morphologic changes in the infected phagocytes induced by mycobacteria. **a** Frequency of characteristic changes. The table reports absolute numbers of incidences and sums of morphological types. **b** Correspondence analysis map for mycobacterial strains and cellular changes. The map was derived from a two-cross tabulation: 1) cellular morphology types, which consisted of four elements (normal shaped macrophages, karyorrhectic, karyopyknotic, and karyolitic phagocytes), 2) six mycobacterial strains (AB1, AB2, AV1, AV2, TB1, TB2). Dispersions of variables produced an asymmetric map, its centroid (point of no correlation) was labeled by a star. All profile points were plotted around this weighted average. A bar plot of variances is shown at the right top part of the map

Characteristic morphological changes in infected macrophages were recorded including cellular shrinkage and nuclear alterations, such as karyopyknosis [which means a shrinkage of the nuclei], karyolysis [lytic disintegration of the nuclei], and karyorrhexis [fragmentation of the nuclei] (Fig. 7). Among all 6 mycobacterial strains, exhibition of karyolysis was 1.58 ± 2.18 %, the range of karyorrhexis was 2.18 ± 2.52 %, while karyopyknosis rate stands at 32.77 ± 4.04 % (Fig. 8a). Interestingly, karyolysis and karyorrhexis were most common with the AV-strains, while absent in TB. The AV-strains cause a small part of karorrhexis, with absence of karyolysis. Among intact infected cells, macrophages accounted for 56.25 ± 4.49 %, and monocytes for 7.23 ± 1.62 %. The differences among strains of the same species were small.

In the case of *M. abscessus* infection, 31.86 % (AB1) and 27.77 % (AB2) of infected cells showed morphological alterations. When *M. tuberculosis* was used (TB1 and TB2), morphological injuries in infected macrophages were evident in 37.44 % and in 37.19 %, respectively. The percentages of morphological alterations caused by AV1 and AV2 strains were 42.11 and 42.81 %. These effects were remarkably higher than those observed in AB and TB. Correspondence analysis reveals association between karyopyknosis and the TB1, TB2, and AV2 strains. Remaining intact structures of phagocytes were observed more often after infection with AB1 and AB2. Karyolitic and karyorrhectic cells show association with the species *M. avium* (Fig. 8c).

Furthermore, results of the TUNEL assay were compared respectively (Fig. 1). Here, the percentages of apoptotic cells in control (MED) and the AB and TB-infected specimens were 3.36 ± 3.34, 8.49 ± 7.64, and 6.15 ± 5.45 %, respectively. In comparison to the other two mycobacterial species, AV strains induced more apoptosis (12.24 ± 9.7 %). Likewise, the mean area per nucleus of living cells was 162.06 units, compared to 73.41 units in dead cells, indicating nuclear shrinkage.

**Fig. 9:**
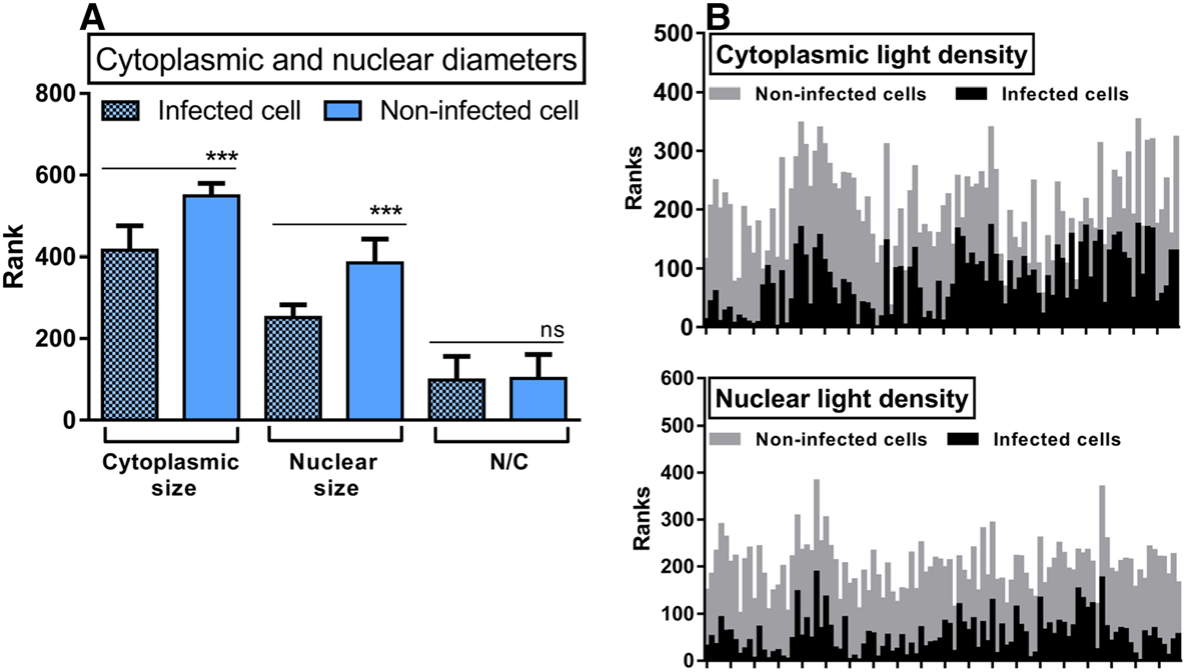
Mycobacterial infections lead to changes in the cellular sizes and light densities. **a** Comparisons of nuclear and cytoplasmic sizes between infected and non-infected cells. Mann–Whitney U-tests were performed, the ranks are given and meanings of p values labeled as asterisks (*** = *p* ≤ 0.001, ns = not significant). **b** Comparisons of cytoplasmic (top) and nuclear (bottom) light densities (LDs) between infected and non-infected cells. Based on the test assumptions Mann–Whitney U tests were chosen and the graphs were created by transformed data of 2 groups, as particularly infected and non-infected cells of nuclear (*N* = 100) and cytoplasmic (*N* = 100) LDs, respectively

While estimating incidences of cellular changes, it became obvious that some infected cells underwent measurable shrinkage characterized by round shape without blebbing and nuclear fragmentation (unlike apoptosis), as well as high condensation of cytoplasm and nucleus (unlike necrosis). In order to characterize this atypical pyknotic change in more detail, 100 normal and 100 infected pyknotic phagocytes were randomly sampled from all cases and species. The above mentioned parameters were assessed to allow a comparison between the groups. (Table 2, Fig. 9). The cytoplasmic and nuclear diameters in the infected pyknotic cells were approximately 1.69 times and 1.75 times smaller (Mann–Whitney *U* test, *p* < 0.001), respectively, than those of non-infected normal cells. Furthermore, their LDs were 4.15 times darker than those of normal cells (Mann–Whitney *U* test, *p* < 0.001). The mean rank of N/C between infected and non-infected cells did not differ (Mann–Whitney *U* test, *p* = 0.642), indicating that the cellular shrinkage involved both compartments. The observation of progressive shrinkage in infected cells with high density of cytosol supports the assumption that this morphological change of infected cells can be a reflection of a kind of cell death induced by mycobacteria. So far, around 15 kinds of cell deaths have been identified. Among them, the above described cellular morphology is in accordance with a self-split cell death, also known as autoschizis.

**Table 2:** Cellular measurements of infected and non-infected cells (Mean ± SD)

Parameters of measurements	Infected cells		Non-infected cells	
	Nucleus	Cytoplasm	Nucleus	Cytoplasm
Light density	−22.83 ± 22.53	43.21 ± 22.59	23.84 ± 17.23	60.71 ± 21.40
Diameter (μm)	4.30 ± 0.77	8.40 ± 1.80	7.53 ± 1.48	14.26 ± 2.31
AM of LD	10.19 ± 18.89		42.28 ± 16.62	
N/C ratio	0.52 ± 0.08		0.53 ± 0.09	

Abbreviation: *AM of LD* arithmetic mean of nuclear and cytoplasmic LD

## Discussion

### Considerations about the proposed model

Infections with pathogenic mycobacteria lead to chronic infections in humans. Numerous in vitro and in vivo animal model systems have been developed and successfully used to gain important insights into this very complex host pathogen interaction. For obvious reasons the detailed analysis of the molecular mechanisms happening during the infection of the human host is very challenging and often hardly possible. In this study we suggest the recently developed STST as a powerful tool to 1) to examine the cross-talk between human host and the infectious agent, 2) to address this in an intact lung parenchyma with its different cell types, 3) to facilitate the analysis of the initial pathogenesis during mycobacterial infections and, 4) to compare different responses induced by virulent and non-virulent strains. The ex vivo STST model allows the investigation of all these demands and has previously been shown to be a source of valuable information of the early processes stages of infections for several pathogens causing lung diseases [34–38, 42].

Histo- and molecular-pathological analyses of the STST samples verified successful mycobacterial infection. Serial sections of a whole block proved that infectious agents diffused sufficiently deep into the tissues. Detailed microscopic evaluation however revealed that mycobacterial infections do not cause any histological damages at the tissue level. By contrast, the study about *Legionella pneumophila* infection by Jager J et al. used the same *ex vivo* model to observe tissue damages and epithelial delaminations [34]. The authors concluded that this harmful extracellular adhesion of *L. pneumophila* facilitates bacterial invasion and replication in recruited macrophages [34].

As assessed by TUNEL assay, STST samples in the current investigation had approximately 7.15 % apoptotic cells. This is a comparably low value (in particular with regard to the presence of mycobacteria) when compared to experimental infection with other bacteria. Feng Xu et al. studied the cellular response to *S. pneumoniae* in the same model system and also performed measurements of apoptosis in lung specimens by TUNEL assay. After pneumococcal infection, a time-dependent increase of apoptosis was observed and compared to the control group (30–50 % versus 4–8 % [24 h], 40–70 % versus 10–15 % [48 h]) [43]. Interestingly, the viability of their control groups was in line with our samples.

### Limitations of the study

Nevertheless, we need to consider that the approach used here has its limitations. Those comprise the relatively high amount of bacteria used together with the chosen infection time of 16 h. We cannot exclude that some of the phenotypes observed may be triggered by the comparably high dose of cell wall components applied. Further studies are necessary to elucidate this point for example by inclusion of heat killed bacteria or inert materials such as beads as further controls. Such controls are commonly used in studies of mycobacterial infections and appropriate studies are currently underway to address this issue. Furthermore, the lack of kinetics is a point that needs to be addressed. The different mycobacterial species might exhibit different dynamics within the tissue culture model. It will be a subject of further studies to address this point together with a comprehensive molecular read out by e.g. transcriptomics. Indeed it would be also desirable to study the infection with lower bacterial doses over a longer period. Due to the declining viability of the tissues in settings of longer than 16 h this is currently not feasible using the STST-model, which is why these points will be addressed by using primary cells.

### Mycobacterial infections and innate immunity

According to the WHO, only 5–10 % of the individuals latently infected with *M. tuberculosis* develop active tuberculosis disease in their lifetime [44]. Also, epidemiological studies showed that only 20–50 % of people with latent tuberculosis exhibit a positive TST skin reaction [45–48]. These TST non-responders may suggest that the individuals remain uninfected because of so far uncharacterized host resistance mechanisms. In this context the immediate clearance of the infection by the innate immune system has been suggested, however this issue is controversially discussed due to the lack of human studies. Hence, with the hypothesis that the innate immune system eliminates mycobacteria, we pointed out functional studies on the initial phase of mycobacterial infection in human lung tissues by using the ex vivo STST model. Firstly, all collected lung specimens of patients were infected and showed that the initial host defense mechanism phagocytosis seems to be actively working. Secondly, we observed that different cell types were infected, including macrophages, neutrophils, monocytes, and pneumocytes-II. At this early stage of infection, an involvement of all these cell types and their cooperation might be the powerful capacity of innate immunity in most individuals. Therefore, each cell type is separately discussed below.

### Mycobacterial infections and innate immunity: Macrophages and monocytes

A total of 900 infected cells were counted for each mycobacterial species, whereby macrophages and monocytes were predominantly infected (85.3 and 6.4 %, after adjustment of cell volume 76.9 and 3.1 %) regardless of the mycobacterial strains. This is in line with the majority of literature reporting that mycobacteria are mainly engulfed by macrophages and circumvent their cellular defense mechanisms to replicate within these cells [9, 49–51]. Correspondence analysis uncovered that monocytes had the tendency to be more likely infected with AB1 and AB2 strains. Furthermore, we observed characteristic cellular changes induced by mycobacteria within phagocytes. These morphologic changes comprise cell shrinkage and nuclear alterations, including karyopyknosis, karyolysis, or karyorrhexis. Based on statistical analyses, we conclude that *M. avium* strains cause significantly more cell injuries and apoptosis. This observation is in accordance with a previous study by Agdestein et al., who characterized the gene expression pattern triggered by two strains of *M. avium* in human primary macrophages [52]. As a result, they demonstrated the induction of pro-apoptotic genes, such as RIPK2, BID, and tBID after infection [52]. Likewise, the correlation between apoptosis and virulence of mycobacterial strains is debated [53]. Moreover, Keane et al. determined that even in comparison among different Mtb strains, attenuated variants of *M. tuberculosis* are associated with macrophage apoptosis, whereas virulent strains inhibit apoptosis to ensure their survival [54].

In the present study, monocytes were recognized by cellular morphology only (cell size, location, shape of nucleus) and not distinguished from macrophages via molecular pathological methods. It had been previously shown that monocytes phagocytizing Mtb secrete pro-inflammatory cytokines and chemokines, such as IL-1β, TNF-α, or IL-8 [55–58]. Since monocytes are freshly recruited host defense cells, these events are obvious contributions to an immune reaction. Impressive findings were observed by Shaw et al. regarding the secretion of IL-10 after phagocytosis of Mtb by human monocytes without blocking autologous IL-8 secretion [59]. The authors concluded that phagocytosis of Mtb by human monocytes displays a specific stimulus to IL-10 secretion [59]. So it is not unlikely that successful removal of mycobacterial infection may be terminated by an early anti-inflammatory activity of monocytes to prevent tissue injury, where the adaptive responses have not been involved at that early time point.

We are convinced that tuberculocidal activity of the innate immunity is based on the phenomenon that humans are not uniformly susceptible to mycobacterial infections. This may operate independently of the acquired immunity via cell death induced during the initial phase of infection. The frequent observation of progressive shrinkage of infected cells with high density might be explained by the presence of a certain kind of cell death, namely “self-split cell death” (also known as autoschizis). Furthermore, autoschizis might be a potential host defense mechanism of eliminating mycobacteria, because its main mechanism is oxidative stress. Thus, organelle-free cytoplasm progressively diminishes through a series of self-excisions due to lipid peroxidation. Also the nucleus becomes smaller; most organelles surround a small intact nucleus in a narrow rim of cytoplasm [60–62]. Interestingly, there are several lines of evidences which indicate the killing of mycobacteria via single members of reactive oxygen species (nitric oxide [63], peroxide [64] and reactive nitrogen intermediates [65]).

### Mycobacterial infections and innate immunity: Neutrophils

Among the six mycobacterial strains, the overall rate of infected neutrophils was 4.7 % (after adjustment 7.1 %). As visualized by correspondence analysis, neutrophils were more often infected with ABs, indicating AB strains may induce the migration of neutrophils more effectively. Neutrophils are essential members of the innate immune system, representing the most abundant type of white blood cells (40–75 %). We observed that the average ratios of infected neutrophils were lower than those of macrophages and monocytes. We showed that AV strains induce the highest rates of apoptosis while the incidences of infected neutrophils are significantly lower in cases of AV strains (*p* = 0.007). Therefore, it seems likely that neutrophils have been more reduced in the AV cases due to apoptosis during incubation. Wolbers et al. reviewed that apoptosis is a dynamic process of variable length depending upon a wide variety of factors, such as the cell type, nature of the inducing agent (i.e., intensity, exposure time), the involved pathway of apoptosis, and the measured parameters [66]. We do not know the exact time course of apoptosis in neutrophils induced after phagocytosis of mycobacteria in our system. It can be quicker than overnight, but should be elucidated in a further separate *in vitro* study. The half-life of circulating neutrophils in the tissue under normal conditions is, however, relatively short (6–8 h) [67] and they are inherently pre-programed to die by inherent apoptosis in order to minimize intracellular propagation and parasitism of pathogens [68–70]. Therefore, it appears that the mycobacterial strategy to survive within host cells by inhibiting apoptosis does not work in neutrophils. Unlike neutrophils, macrophages exhibit a longer life span and provide an opportunity for mycobacteria to shelter since they established strategies to circumvent phagosomal maturation such as Mtb [71]. On the other hand, it has been observed that mycobacteria in apoptotic bodies were eliminated since they were not able to inhibit the phagolysosome fusion from the inside, when they are engulfed by fresh phagocytes [72].

Besides of apoptosis, there is some evidence showing an intrinsic mycobactericidal capacity of neutrophils via both oxidase-dependent [73–75] and independent [76–79] mechanisms. Whether this is associated with a distinct set of receptors used for recognition and phagocytosis which trigger different intracellular cascades is currently not known. In addition, epidemiological reports suggest that neutrophils may contribute substantially to the innate resistance of *M. tuberculosis* infections. Particularly, the prevalence of tuberculosis among people of African origin is known to be about as twice as high as observed in Caucasian donors [80]. Lower neutrophil counts and lower circulating concentrations of neutrophil derived factors HNP1–3 and lipocalin 2 in these individuals when compared to people of South Asian and Caucasian origin, demonstrate the importance of neutrophils for the prevention of tuberculosis [78].

### Mycobacterial infections and innate immunity: Pneumocytes-II

It has been repeatedly described that inhaled infected droplets with mycobacteria reach the alveolar space and are mainly engulfed by alveolar macrophages. After morphometric correction, we observed 13 % of the mycobacterial infections in pneumocytes-II in our human ex vivo model system. Compared to AV and AB, infected pneumocytes-II were more common in infections with TB strains (22.2 %), as visualized by correspondence analysis. This finding is unexpected but demonstrates the role of epithelial cells in the initial phase of Mtb-infection in the human lung. According to the differences in the rates of infected pneumocytes-II by the different mycobacterial species, we hypothesize that NTM (AB and AV strains) induce more cell death also to pneumocytes-II, and the cell wall features of Mtb could exhibit a higher affinity to the receptors which facilitate phagocytosis in pneumocytes-II compared to those of NTM (AB and AV). On the other hand, Feng Xu et al. studied the inflammatory response of *S. pneumoniae* in a similar model system and observed that after 24 h incubation, *S. pneumoniae* was predominantly detected in alveolar macrophages compared to pneumocytes-II [80–90 % versus 15–30 %]. Interestingly, the authors also noticed that 48 h-stimulation increased the infection rates of pneumocytes-II [43]. It seems to be that the internalization by pneumocytes-II is slow, since they are considered as non-professional phagocytes. Further studies in our department are currently ongoing using primary pneumocytes-II as an infection model.

## Conclusion

In this study we demonstrate that the STST model can be successfully employed in the analysis of mycobacterial infection of human intact lung parenchyma. We observed at the cellular level, that different cell types (macrophages, monocytes, neutrophils, and pneumocytes-II) were infected. In these infected cells, characteristic morphologic changes (cell shrinkage and nuclear alterations) were observed, most likely reflecting cell death. The infection frequency of a given cell type and the infection dependent extent of morphologic changes were significantly associated with mycobacterial species. This adds to the current understanding of primary infection of mycobacteria. Especially, the involvement of several cell types, their cooperation, cell death dependent and cell death independent effector functions do contribute to the overall capacity of a human individual to efficiently limit the growth of mycobacteria upon exposure. Thus, the STST model provides a valuable novel tool to address the complex mechanisms of innate effector faction in the very early onset of TB disease. Further studies are ongoing in order to characterize the interaction of pathogenic mycobacteria with the human host at the subcellular, cellular and supracellular organ level.

## Abbreviations

BMB: Biomaterialbank
CFU: Colony forming units
DAPI: 4’-6-DiAmidino-2-PhenylIndole
FCS: Fetal calf serum
H&E: Hematoxylin and Eosin
HBSS: Hanks’ balanced salt solution
HOPE: HEPES-glutamic acid buffer mediated organic solvent protection effect
IHC: immunohistochemistry
LJ: Löwenstein–Jensen medium
LD: light density
MOI: multiplicity of infection
MTT: (4,5-diMethylThiazol-2-yl)-2,5-diphenylTetrazolium bromide
Mtb: *M. tuberculosis*
N/C ratio: A ratio of the nuclear size to the cytoplasmic size
NTM: Non-tuberculous mycobacteria
PBS: Phosphate-buffered saline
PCR: Polymerase chain reaction
STST: Short-term stimulation of tissues
TUNEL: Terminal deoxynucleotidyl transferase dUTP Nick End Labeling
ZN: Ziel Neelsen

## References

[1] Organization WH: Global Tuberculosis Report 2014. World Health Organization; 2015.

[2] Griffith DE, Aksamit T, Brown-Elliott BA, Catanzaro A, Daley C, Gordin F (2007). An official ATS/IDSA statement: diagnosis, treatment, and prevention of nontuberculous mycobacterial diseases. Am J Respir Crit Care Med.

[3] van Ingen J, Bendien SA, de Lange WC, Hoefsloot W, Dekhuijzen PN, Boeree MJ (2009). Clinical relevance of non-tuberculous mycobacteria isolated in the Nijmegen-Arnhem region, The Netherlands. Thorax.

[4] Gupta UD, Katoch VM (2005). Animal models of tuberculosis. Tuberculosis (Edinb).

[5] Ulrichs T, Kaufmann SH (2002). Mycobacterial persistence and immunity. Front Biosci.

[6] Shen Y, Zhou D, Qiu L, Lai X, Simon M, Shen L (2002). Adaptive immune response of Vgamma2Vdelta2+ T cells during mycobacterial infections. Science.

[7] Shi L, Ryan GJ, Bhamidi S, Troudt J, Amin A, Izzo A (2014). Isolation and purification of Mycobacterium tuberculosis from H37Rv infected guinea pig lungs. Tuberculosis (Edinb).

[8] Flynn JL (2006). Lessons from experimental Mycobacterium tuberculosis infections. Microbes Infect.

[9] Actor J, Hunter R, Jagannath C, Zander D, Popper H, Jagirdar J, Haque A, Cagle P, Barrios R (2008). Immunopathology of Tuberculosis. Molecular Pathology of Lung Diseases. Volume 1.

[10] Rivero-Lezcano OM (2013). In vitro infection of human cells with Mycobacterium tuberculosis. Tuberculosis (Edinb).

[11] Danelishvili L, McGarvey J, Li YJ, Bermudez LE (2003). Mycobacterium tuberculosis infection causes different levels of apoptosis and necrosis in human macrophages and alveolar epithelial cells. Cell Microbiol.

[12] Lee J, Remold HG, Leong MH, Kornfeld H (2006). Macrophage apoptosis in response to high intracellular burden of Mycobacterium tuberculosis is mediated by a novel caspase-independent pathway. J Immunol.

[13] Crowle AJ, May M (1981). Preliminary demonstration of human tuberculoimmunity in vitro. Infect Immun.

[14] Steele J, Flint KC, Pozniak AL, Hudspith B, Johnson MM, Rook GA (1986). Inhibition of virulent Mycobacterium tuberculosis by murine peritoneal macrophages and human alveolar lavage cells: the effects of lymphokines and recombinant gamma interferon. Tubercle.

[15] Brown AE, Holzer TJ, Andersen BR (1987). Capacity of human neutrophils to kill Mycobacterium tuberculosis. J Infect Dis.

[16] Peterson PK, Gekker G, Hu S, Sheng WS, Anderson WR, Ulevitch RJ (1995). CD14 receptor-mediated uptake of nonopsonized Mycobacterium tuberculosis by human microglia. Infect Immun.

[17] Stokes RW, Doxsee D (1999). The receptor-mediated uptake, survival, replication, and drug sensitivity of Mycobacterium tuberculosis within the macrophage-like cell line THP-1: a comparison with human monocyte-derived macrophages. Cell Immunol.

[18] Rockett KA, Brookes R, Udalova I, Vidal V, Hill AV, Kwiatkowski D (1998). 1,25-Dihydroxyvitamin D3 induces nitric oxide synthase and suppresses growth of Mycobacterium tuberculosis in a human macrophage-like cell line. Infect Immun.

[19] Caccamo N, Milano S, Di Sano C, Cigna D, Ivanyi J, Krensky AM (2002). Identification of epitopes of Mycobacterium tuberculosis 16-kDa protein recognized by human leukocyte antigen-A*0201 CD8(+) T lymphocytes. J Infect Dis.

[20] Shepard CC (1957). Growth characteristics of tubercle bacilli and certain other mycobacteria in HeLa cells. J Exp Med.

[21] Bermudez LE, Goodman J (1996). Mycobacterium tuberculosis invades and replicates within type II alveolar cells. Infect Immun.

[22] Mackaness GB (1954). The growth of tubercle bacilli in monocytes from normal and vaccinated rabbits. Am Rev Tuberc.

[23] Suter E (1952). The multiplication of tubercle bacilli within normal phagocytes in tissue culture. J Exp Med.

[24] Patterson RJ, Youmans GP (1970). Multiplication of Mycobacterium tuberculosis Within Normal and “Immune” Mouse Macrophages Cultivated With and Without Streptomycin. Infect Immun.

[25] Lamhamedi-Cherradi S, de Chastellier C, Casanova JL (1999). Growth of Mycobacterium bovis, Bacille Calmette-Guerin, within human monocytes-macrophages cultured in serum-free medium. J Immunol Methods.

[26] Ernst JD (1998). Macrophage receptors for Mycobacterium tuberculosis. Infect Immun.

[27] Wallis RS, Vinhas S, Janulionis E (2009). Strain specificity of antimycobacterial immunity in whole blood culture after cure of tuberculosis. Tuberculosis (Edinb).

[28] Bermudez LE, Sangari FJ, Kolonoski P, Petrofsky M, Goodman J (2002). The efficiency of the translocation of Mycobacterium tuberculosis across a bilayer of epithelial and endothelial cells as a model of the alveolar wall is a consequence of transport within mononuclear phagocytes and invasion of alveolar epithelial cells. Infect Immun.

[29] Bonay M, Bouchonnet F, Pelicic V, Lagier B, Grandsaigne M, Lecossier D (1999). Effect of stimulation of human macrophages on intracellular survival of Mycobacterium bovis Bacillus Calmette-Guerin. Evaluation with a mycobacterial reporter strain. Am J Respir Crit Care Med.

[30] Martineau AR, Wilkinson KA, Newton SM, Floto RA, Norman AW, Skolimowska K (2007). IFN-gamma- and TNF-independent vitamin D-inducible human suppression of mycobacteria: the role of cathelicidin LL-37. J Immunol.

[31] Garg SK, Volpe E, Palmieri G, Mattei M, Galati D, Martino A (2004). Sphingosine 1-phosphate induces antimicrobial activity both in vitro and in vivo. J Infect Dis.

[32] Kusner DJ, Adams J (2000). ATP-induced killing of virulent Mycobacterium tuberculosis within human macrophages requires phospholipase D. J Immunol.

[33] Morrison J, Pai M, Hopewell PC (2008). Tuberculosis and latent tuberculosis infection in close contacts of people with pulmonary tuberculosis in low-income and middle-income countries: a systematic review and meta-analysis. Lancet Infect Dis.

[34] Jager J, Marwitz S, Tiefenau J, Rasch J, Shevchuk O, Kugler C (2014). Human lung tissue explants reveal novel interactions during Legionella pneumophila infections. Infect Immun.

[35] Harrison F, Muruli A, Higgins S, Diggle SP (2014). Development of an ex vivo porcine lung model for studying growth, virulence, and signaling of Pseudomonas aeruginosa. Infect Immun.

[36] Szymanski KV, Toennies M, Becher A, Fatykhova D, N’Guessan PD, Gutbier B (2012). Streptococcus pneumoniae-induced regulation of cyclooxygenase-2 in human lung tissue. Eur Respir J.

[37] Rupp J, Droemann D, Goldmann T, Zabel P, Solbach W, Vollmer E (2004). Alveolar epithelial cells type II are major target cells for C. pneumoniae in chronic but not in acute respiratory infection. FEMS Immunol Med Microbiol.

[38] Droemann D, Rupp J, Rohmann K, Osbahr S, Ulmer AJ, Marwitz S (2010). The TGF-beta-pseudoreceptor BAMBI is strongly expressed in COPD lungs and regulated by nontypeable Haemophilus influenzae. Respir Res.

[39] Shevchuk O, Abidi N, Klawonn F, Wissing J, Nimtz M, Kugler C (2014). HOPE-Fixation of Lung Tissue Allows Retrospective Proteome and Phosphoproteome Studies. J Proteome Res.

[40] Olert J, Wiedorn KH, Goldmann T, Kuhl H, Mehraein Y, Scherthan H (2001). HOPE fixation: a novel fixing method and paraffin-embedding technique for human soft tissues. Pathol Res Pract.

[41] Fellenberg K, Hauser NC, Brors B, Neutzner A, Hoheisel JD, Vingron M (2001). Correspondence analysis applied to microarray data. Proc Natl Acad Sci U S A.

[42] Lang DS, Droemann D, Schultz H, Branscheid D, Martin C, Ressmeyer AR (2007). A novel human ex vivo model for the analysis of molecular events during lung cancer chemotherapy. Respir Res.

[43] Xu F, Droemann D, Rupp J, Shen H, Wu X, Goldmann T (2008). Modulation of the inflammatory response to Streptococcus pneumoniae in a model of acute lung tissue infection. Am J Respir Cell Mol Biol.

[44] Organization WH: Global Tuberculosis Report 2013. World Health Organization; 2014

[45] O’Garra A, Redford PS, McNab FW, Bloom CI, Wilkinson RJ, Berry MP (2013). The immune response in tuberculosis. Annu Rev Immunol.

[46] Jereb J, Etkind SC, Joglar OT, Moore M, Taylor Z (2003). Tuberculosis contact investigations: outcomes in selected areas of the United States, 1999. Int J Tuberc Lung Dis.

[47] Marks SM, Taylor Z, Qualls NL, Shrestha-Kuwahara RJ, Wilce MA, Nguyen CH (2000). Outcomes of contact investigations of infectious tuberculosis patients. Am J Respir Crit Care Med.

[48] Styblo K (1980). Recent advances in epidemiological research in tuberculosis. Adv Tuberc Res.

[49] Vinay Kumar AKA, Fausto N, Aster J (2010). Robbins and Cotran: Pathologic Basis of Disease.

[50] Robert J. Mason VCB, Thomas Martin, Talmadge E King, Jr., Schraufnagel, John F. Murray, FRCP, Jay A. Nadel, Jay A. Nadel, : Murray and Nadel’s Textbook of Respiratory Medicine. Saunders; 2010.

[51] Ryan K, Ray CG, Ahmad N, Drew WL, Plorde J (2009). Sherris Medical Microbiology.

[52] Agdestein A, Jones A, Flatberg A, Johansen TB, Heffernan IA, Djonne B (2014). Intracellular growth of Mycobacterium avium subspecies and global transcriptional responses in human macrophages after infection. BMC Genomics.

[53] Lee J, Hartman M, Kornfeld H (2009). Macrophage apoptosis in tuberculosis. Yonsei Med J.

[54] Keane J, Remold HG, Kornfeld H (2000). Virulent Mycobacterium tuberculosis strains evade apoptosis of infected alveolar macrophages. J Immunol.

[55] Wallis RS, Amir-Tahmasseb M, Ellner JJ (1990). Induction of interleukin 1 and tumor necrosis factor by mycobacterial proteins: the monocyte western blot. Proc Natl Acad Sci U S A.

[56] Zhang Y, Doerfler M, Lee TC, Guillemin B, Rom WN (1993). Mechanisms of stimulation of interleukin-1 beta and tumor necrosis factor-alpha by Mycobacterium tuberculosis components. J Clin Invest.

[57] Fulton SA, Cross JV, Toossi ZT, Boom WH (1998). Regulation of interleukin-12 by interleukin-10, transforming growth factor-beta, tumor necrosis factor-alpha, and interferon-gamma in human monocytes infected with Mycobacterium tuberculosis H37Ra. J Infect Dis.

[58] Friedland JS, Remick DG, Shattock R, Griffin GE (1992). Secretion of interleukin-8 following phagocytosis of Mycobacterium tuberculosis by human monocyte cell lines. Eur J Immunol.

[59] Shaw TC, Thomas LH, Friedland JS (2000). Regulation of IL-10 secretion after phagocytosis of Mycobacterium tuberculosis by human monocytic cells. Cytokine.

[60] Gilloteaux J, Jamison JM, Arnold D, Summers JL (2001). Autoschizis: another cell death for cancer cells induced by oxidative stress. Ital J Anat Embryol.

[61] Gilloteaux J, Jamison JM, Arnold D, Ervin E, Eckroat L, Docherty JJ (1998). Cancer cell necrosis by autoschizis: synergism of antitumor activity of vitamin C: vitamin K3 on human bladder carcinoma T24 cells. Scanning.

[62] Pais V, Danaila L, Pais E (2012). A comparative ultrastructural study of a new type of autoschizis versus a survival cellular mechanism that involves cell membranes of cerebral arteries in humans. Ultrastruct Pathol.

[63] Esquivel-Solis H, Vallecillo AJ, Benitez-Guzman A, Adams LG, Lopez-Vidal Y, Gutierrez-Pabello JA (2013). Nitric oxide not apoptosis mediates differential killing of Mycobacterium bovis in bovine macrophages. PLoS One.

[64] Mendoza-Aguilar MD, Arce-Paredes P, Aquino-Vega M, Rodríguez-Martínez S, Rojas-Espinosa O (2013). Fate of Mycobacterium tuberculosis in peroxidase-loaded resting murine macrophages. International Journal of Mycobacteriology.

[65] Chan J, Xing Y, Magliozzo RS, Bloom BR (1992). Killing of virulent Mycobacterium tuberculosis by reactive nitrogen intermediates produced by activated murine macrophages. J Exp Med.

[66] Wolbers F, Buijtenhuijs P, Haanen C, Vermes I (2004). Apoptotic cell death kinetics in vitro depend on the cell types and the inducers used. Apoptosis.

[67] Fox S, Leitch AE, Duffin R, Haslett C, Rossi AG (2010). Neutrophil apoptosis: relevance to the innate immune response and inflammatory disease. J Innate Immun.

[68] McCracken JM, Allen LA (2014). Regulation of human neutrophil apoptosis and lifespan in health and disease. J Cell Death.

[69] Young B, Stewart W, O’Dowd G (2011). Wheater’s Basic Pathology: A Text, Atlas and Review of Histopathology.

[70] Summers C, Rankin SM, Condliffe AM, Singh N, Peters AM, Chilvers ER (2010). Neutrophil kinetics in health and disease. Trends Immunol.

[71] Vergne I, Chua J, Singh SB, Deretic V (2004). Cell biology of mycobacterium tuberculosis phagosome. Annu Rev Cell Dev Biol.

[72] Bocchino M, Galati D, Sanduzzi A, Colizzi V, Brunetti E, Mancino G (2005). Role of mycobacteria-induced monocyte/macrophage apoptosis in the pathogenesis of human tuberculosis. Int J Tuberc Lung Dis.

[73] Yang CT, Cambier CJ, Davis JM, Hall CJ, Crosier PS, Ramakrishnan L (2012). Neutrophils exert protection in the early tuberculous granuloma by oxidative killing of mycobacteria phagocytosed from infected macrophages. Cell Host Microbe.

[74] Bogdan C, Rollinghoff M, Diefenbach A (2000). Reactive oxygen and reactive nitrogen intermediates in innate and specific immunity. Curr Opin Immunol.

[75] Winterbourn CC (2008). Reconciling the chemistry and biology of reactive oxygen species. Nat Chem Biol.

[76] Jones GS, Amirault HJ, Andersen BR (1990). Killing of Mycobacterium tuberculosis by neutrophils: a nonoxidative process. J Infect Dis.

[77] Kisich KO, Higgins M, Diamond G, Heifets L (2002). Tumor necrosis factor alpha stimulates killing of Mycobacterium tuberculosis by human neutrophils. Infect Immun.

[78] Martineau AR, Newton SM, Wilkinson KA, Kampmann B, Hall BM, Nawroly N (2007). Neutrophil-mediated innate immune resistance to mycobacteria. J Clin Invest.

[79] Majeed M, Perskvist N, Ernst JD, Orselius K, Stendahl O (1998). Roles of calcium and annexins in phagocytosis and elimination of an attenuated strain of Mycobacterium tuberculosis in human neutrophils. Microb Pathog.

[80] Stead WW, Senner JW, Reddick WT, Lofgren JP (1990). Racial differences in susceptibility to infection by Mycobacterium tuberculosis. N Engl J Med.

